# Modeling the Propagation of Mobile Phone Virus under Complex Network

**DOI:** 10.1155/2014/207457

**Published:** 2014-07-15

**Authors:** Wei Yang, Xi-liang Wei, Hao Guo, Gang An, Lei Guo, Yu Yao

**Affiliations:** ^1^Software College, Northeastern University, Shenyang 110819, China; ^2^College of Information Science and Engineering, Northeastern University, Shenyang 110819, China; ^3^Quality and Technology Department, Liaoning Provincial Institute of Measurement, Shenyang 110004, China; ^4^Information Center, Panjin Finance Bureau, Panjin 124000, China; ^5^Key Laboratory of Medical Image Computing, Northeastern University, Ministry of Education, Shenyang 110819, China

## Abstract

Mobile phone virus is a rogue program written to propagate from one phone to another, which can take control of a mobile device by exploiting its vulnerabilities. In this paper the propagation model of mobile phone virus is tackled to understand how particular factors can affect its propagation and design effective containment strategies to suppress mobile phone virus. Two different propagation models of mobile phone viruses under the complex network are proposed in this paper. One is intended to describe the propagation of user-tricking virus, and the other is to describe the propagation of the vulnerability-exploiting virus. Based on the traditional epidemic models, the characteristics of mobile phone viruses and the network topology structure are incorporated into our models. A detailed analysis is conducted to analyze the propagation models. Through analysis, the stable infection-free equilibrium point and the stability condition are derived. Finally, considering the network topology, the numerical and simulation experiments are carried out. Results indicate that both models are correct and suitable for describing the spread of two different mobile phone viruses, respectively.

## 1. Introduction

More and more rogue programs called mobile phone virus, which can take control of a mobile device by exploiting its vulnerabilities, can be written to propagate from one phone to another. Security issues of mobile phones have become increasingly prominent. Though attacks from the mobile phone virus have not caused greater damage up to now, it is just a matter of time before it breaks out [1]. The large population of mobile users and the wide coverage of mobile communication network [2] create a breeding ground for the propagation of mobile phone virus. The propagation of mobile phone virus may be more potentially destructive than the computer virus. In this regard, mobile phone virus encounters a similar situation of Internet worms, so it is necessary to research the propagation behavior of mobile phone virus in the real world and design effective containment strategies to suppress them.

The usual ways for mobile phone virus to propagate include multimedia messaging service (MMS) [3] interface and e-mail services on that mobile phone. MMS messages are intended to contain media content such as photos, audios, and videos, but they can also contain infected malicious codes [4]. One noteworthy example is Commwarrior [5], which is the first mobile phone virus that can propagate via MMS. It searches for phone number through a user's local address book and sends MMS messages containing infected files to other users in the address book. It is an easy way for mobile phone virus to carry out because people are more likely to open and download the contents that they received from their friends. So the mobile phone virus could be sent out in just one click and travel to any mobiles all over the world with a larger chance of success in propagation [4].

The mobile phone virus is in the time of high-speed development. In the present, it only reproduces and propagates by tricking mobile phone users, called user-tricking virus, but does not spread automatically. According to the development rules of the computer virus, the future trend of the mobile phone virus is that it can propagate by exploiting vulnerabilities existing in mobile phone operating systems or application software. That is, the propagation of such mobile phone virus called vulnerability-exploiting virus can be realized by itself without human participants.

The possible path through which mobile phone virus spreads depends on the social relationship of a user by exploiting the local address book or recent call records. Communications based on social network provide the environment for the spread of the mobile phone virus. And the social network will greatly influence the spread of the mobile phone virus. Recently some researchers have studied the structures of social network topologies [6–8]. They found that all of these networks are complex network and they have power-law degree distributions. Existing work on studying mobile phone virus [9, 10] does not take into account the capability of mobile phone virus to spread under complex network. Consequently this paper focuses on researching the behavior of the user-tricking virus in the present and the vulnerability-exploiting virus in the future combining the topology of the complex network.

Many particular factors can affect the propagation of mobile phone virus and its behavior is very complicated depending on the social relationship of mobile phone users. So an extremely fundamental and effective way to study the mobile phone virus is using the epidemiological models. Epidemiological models are the usual method used to understand and predict the propagation of Internet worms by many researchers [11–20].

The mobile phone virus has some commons with the Internet worms. As the behavior of mobile phone virus is more complex than that of Internet worms, it is necessary to construct a new model for virus propagation. Due to the characteristic of exponential propagation exhibited by mobile phone virus through complex network, it is challenging to model the user-tricking and vulnerability-exploiting mobile phone virus.

Through above observations, this paper models the propagation of mobile phone virus considering the characteristics of mobile phone viruses and the network topology structure. The contributions of this paper are as follows.

- Two different propagation models of mobile phone viruses under the complex network are proposed in this paper. One is intended to describe the propagation of user-tricking virus, and the other is to describe the propagation of the vulnerability-exploiting virus.
- A detailed analysis is conducted to analyze the propagation models. Through analysis, the stable infection-free equilibrium point and the stability condition are derived.
- Considering the network topology, the numerical and simulation experiments are carried out. Results indicate that both models are correct and suitable for describing the spread of two different mobile phone viruses, respectively.

The rest of this paper is organized as follows.  Section 2 presents related work about modeling the mobile phone virus.  Section 3 conducts and analyzes the mobile phone virus SIS propagation model (M-SIS) and obtains the stability condition and the infection-free equilibrium point.  Section 4 proposes the propagation model of vulnerability-exploiting mobile phone virus, which is called the mobile phone virus SIR propagation model (M-SIR).  Section 5 describes the constructing process of complex network which is used to simulate the spread of the mobile phone virus.  Section 6 concludes the paper and provides future research directions.

## 2. Related Work

Mobile phone users communicate and share files with their friends and they also take part in some activities or join groups online [21]. These characteristics give hackers the opportunities to attack mobile users. As a result, the mobile phone virus can spread quickly. More and more researchers pay attention to the area of mobile phone virus. But the research on the mobile phone virus is just in the beginning stage. Fundamental research works on it have been gradually carried out in order to raise the security awareness among users.

Leavitt lists some mobile phone viruses, such as Cabir, Skulls, and Mosquito and points out increasing virus attacks to mobile phones [1]. But he deems that a method always can be found to cope with the security issue caused by the mobile phone virus. Dwan takes the mobile phone virus “Cabir” as an example to emphasize the lack of security mechanism and expects to take certain security measures from both mobile phones' software and hardware [22]. Jamaluddin et al. state the damage of the mobile phone virus and predict that the mobile phone virus will develop along the path of the computer virus and cause serious security problems [23]. Dagon et al. describe the security threat with which mobile users are faced and propose several security advices to mobile users [24].

With the popularity of Android platform based mobile phones, more and more attention is paid to the protection of mobile phones. Zhang et al. propose a browser-free multilevel smart phone privacy protection system by means of short message system [25]. Based on the specific network of short message, Jin et al. proposed an epidemic model of mobile phone virus based on the efficiency of immunization to reveal the spreading rule of mobile phone virus [26].

Based on the similarity between a malicious worm and a biological virus, some epidemic models representing worm propagation were presented to depict the propagation of worms, for example, SIS model (susceptible-infectious-susceptible) and SIR model (susceptible-infectious-recovered) [27]. Yao et al. research the worm propagation model by considering the time delay [28]. They found that time delay may lead to Hopf bifurcation phenomenon which will make the worm propagation system unstable and uncontrollable.

Propagation models and the stability of mobile phone virus become an attractive research field in recent years since it facilitates worm prediction, detection, analysis and prevention, and so forth. There have been some models to simulate the mobile phone virus propagation.

Wang et al. modeled the mobility of mobile phone users in order to study the fundamental spreading patterns that characterize a mobile virus outbreak [29]. Their results explain the lack of a major mobile virus breakout so far and predict that once a mobile operating system's market share reaches the phase transition point, viruses will pose a serious threat to mobile communications. Zheng et al. analyze the communication of Bluetooth between mobile users and put forward a propagation model of the mobile phone virus which spreads through Bluetooth technique [30]. Xuetao et al. propose and evaluate a SI_1_I_2_S, a competition model that describes the spread of two mutually exclusive mobile viruses across heterogeneous composite networks [31].

Existing propagation models of mobile phone virus focus on the specific kind of virus. This kind of virus spreads using Bluetooth or short message, which is completely different from the virus spreading using MMS.

Mobile phone virus that spreads using MMS typically exploits the social network of users to propagate from one mobile device to another. So the topology of network is a key factor for this kind of mobile virus using MMS to propagation. As far as I know no one has studied the propagation model of this kind virus. So considering the characteristics of mobile phone virus and the social network relationship, two different propagation models of mobile phone viruses under the complex network are proposed in this paper to understand how particular factors can affect their propagation and design effective containment strategies to suppress mobile phone virus.

## 3. Modeling the Propagation of the User-Tricking Mobile Phone Virus

### 3.1. M-SIS Model

The user-tricking virus only reproduces and propagates by tricking when mobile phone users are in just one click. In this regard, the following assumption is made that the propagation path of a mobile virus can be approximated by the social network of mobile devices. Given that a user A has a higher probability to open and download a message from B with whom he periodically exchanges messages, the pair of users, A-B, would be considered more vulnerable. In contrast, if user A does not exchange messages with user C, the user A is unlikely to be infected by a mobile phone virus sent by C and hence the pair of A-C is considered less likely to be included in the propagation path of the mobile virus. This kind of virus is now prevailing on current mobile phone system and is difficult to kill completely. It will mislead users to install and then execute a norm application. Even if it removed, it can do the same thing with another guise again.

An undirected graph *G* = (*V*, *E*) consisting of a set of vertices *V* and a set of edges *E* is used to denote mobile phone communication system. Each vertex *u* ∈ *V* denotes a mobile in the cellular network and each edge *e*(*u*, *v*) denotes that at least one traffic flow was exchanged between mobiles *u* and *v*. Let *d*_*i*_ denote the degree of any vertex *i* ∈ *V*. According to the spread property of user-tricking mobile phone virus, the epidemic states of a mobile are divided as follows: susceptible state (*S*) and infectious state (*I*).

Susceptible state (*S*): nodes have not been infected by any user-tricking virus but are prone to infection.

Infectious state (*I*): nodes have been infected by user-tricking virus and they may infect some mobiles in state S.

Mobile users who have larger groups of friends in social network tend to appear in the contact list of many others [32]. Different nodes with different vertex's degree have different behavior to the spread of mobile phone virus. So it is necessary to study the propagation process of mobile nodes with different node's degree.

According to the nodes' degrees, these vertices in the undirected graph are classified into *n* kinds of nodes. The nodes with the same degree belong to a class. Let *N*_*i*_ denote the number of the *i*th kinds of nodes while the value of *i* ranges from 1 to *n*. It is assumed that there are totally *N* mobiles deployed in the communication network. So the sum of *n* kinds of mobiles is expressed as follows:

(1) $$\begin{array}{c} N=N_{1}+N_{2}+\cdots +N_{i}+N_{n}. \end{array}$$

Let *S*_*i*_(*t*) represent the number of the *i*th kinds of mobiles in the susceptible state at *t* time. *I*_*i*_(*t*) is defined as the number of the *i*th kinds of mobiles in the infected state at *t* time. So the number of the *i*th kinds of mobiles can be obtained as follows:

(2) $$\begin{array}{c} S_{i}\left(t\right)+I_{i}\left(t\right)=N_{i}. \end{array}$$

In the social network, a mobile can leave or join the network randomly. So the “death” of a mobile is defined to refer to the fact that a phone drops out of the network for certain reason, such as breakdown. The “birth” means that the network adds a new mobile. But it is assumed that the system is a closed system and the number of “dead” rates of mobile is the same as that of the “birth” one.

**Figure 1:**
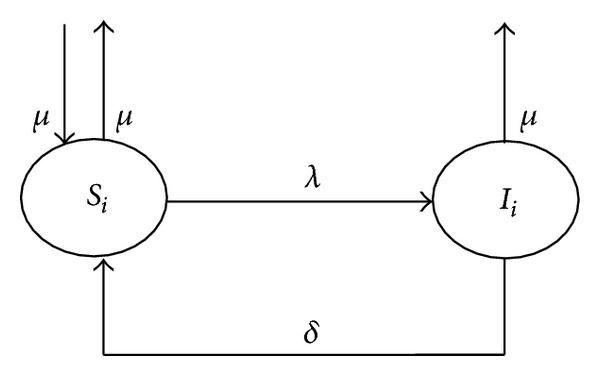
State transition graph of the *i*th kind of mobiles in M-SIS model.

The propagation modeling of user-tricking mobile phone virus under complex network called M-SIS model is proposed, which means mobile phone virus SIS (susceptible-infectious-susceptible) propagation model. In the M-SIS model, M represents mobile phone, and *S* stands for the susceptible state while *I* stands for the infectious state. The state transforming process of any kind of mobiles in M-SIS model is illustrated in Figure 1.

A node may change its states as follows.

Node *i* of any kind can transit to the infectious state if it is at the susceptible state. The infection probability, also called contact infection rate, is presented by *λ*.

A mobile is not permanently immune against the virus and has a risk of reinfection. So a mobile at the infectious state can kill the virus and recover to the susceptible state. The infection recovery rate is presented by *δ*.

To maintain the balance of the network system, the “death” rate and the “birth” rate are all *μ*. The “new born” mobiles are all in the susceptible state.

The description of related parameters in M-SIS model is showed in Table 1.

Based on the above analysis and compartment model of *i*th kind presented in Figure 1, given a topology of a mobile communication network, the number of susceptible and infected nodes of the *i*th kind at time *t* in the M-SIS model can be formulated by the equations as follows:

(3) $$\begin{array}{c} \frac{dS_{i}\left(t\right)}{dt}=\mu N_{i}-\mu S_{i}\left(t\right)-\lambda k_{i}S_{i}\left(t\right)\Theta \left(t\right)+\delta I_{i}\left(t\right),\\ \frac{dI_{i}\left(t\right)}{dt}=\lambda k_{i}S_{i}\left(t\right)\Theta \left(t\right)-\mu I_{i}\left(t\right)-\delta I_{i}\left(t\right). \end{array}$$

In (3), *k*_*i*_ is the degree of the *i*th kind of mobile phone nodes, where *i* = 1,2,…, *n*. Θ(*t*) is the infected probability that any of neighbor nodes of one mobile phone node and the expression of Θ(*t*) are as

(4) $$\begin{array}{c} \Theta \left(t\right)=\frac{\sum_{i=1}^{n}k_{i}P\left(k_{i}\right)I_{i}\left(t\right)}{\langle k\rangle }. \end{array}$$

In (4), 〈*k*〉 means the average degree of nodes in the network, which can be expressed as

(5) $$\begin{array}{c} \langle k\rangle =\sum k_{i}P\left(k_{i}\right), \end{array}$$

where *P*(*k*_*i*_) is the probability density of nodes with the degree *k*_*i*_. So the differential equations of the M-SIS model can be concluded as the following equation:

(6) $$\begin{array}{c} \frac{dS\left(t\right)}{dt}=\overset{n}{\underset{i=1}{\sum }}\frac{dS_{i}\left(t\right)}{dt},\\ \frac{dI\left(t\right)}{dt}=\overset{n}{\underset{i=1}{\sum }}\frac{dI_{i}\left(t\right)}{dt}. \end{array}$$

### 3.2. Infection-Free Equilibrium Point

The infection-free equilibrium refers to the fact that the mobile virus gets removed and the number of infected mobiles remains 0. To derive the infection-free equilibrium point, let both *dS*_*i*_(*i*)/*dt* and *dI*_*i*_(*i*)/*dt* be equal to 0, and the following expression is obtained as

(7) $$\begin{array}{c} \mu N_{i}-\mu S_{i}\left(t\right)-\lambda k_{i}S_{i}\left(t\right)\Theta \left(t\right)+\delta I_{i}\left(t\right)=0,\\ \lambda k_{i}S_{i}\left(t\right)\Theta \left(t\right)-\mu I_{i}\left(t\right)-\delta I_{i}\left(t\right)=0 \end{array}$$

When *I*_*i*_ = 0, *S*_*i*_(*t*) and *I*_*i*_(*t*) can be calculated as follows, where *i* = 1,2,…, *n*:

(8) $$\begin{array}{c} S_{i}\left(t\right)=N_{i},\\ I_{i}\left(t\right)=0. \end{array}$$

The number of the *i*th kinds of mobile phones in the susceptible state is *N*_*i*_, while that of the *i*th kinds of mobile phones in the infectious state is 0. The infection-free equilibrium point of the mobile phone virus propagation system under the M-SIS model is thus *E*_0_*(*N*_1_, 0, *N*_2_, 0,…, *N*_*n*_, 0).

### 3.3. Stability of the Infection-Free Equilibrium

Though the user-tricking virus is difficult to completely kill and mobiles are not permanently immune, it is ensured that the number of infected mobiles can dynamically remain 0. It means that the infection-free equilibrium can be achieved. Its stability for the propagation system of the mobile phone virus will be discussed.

**Theorem 1 .**

If the basic reproduction number *R*_0_ < 1, the propagation system under the M-SIS model of mobile phone virus will stabilize at the infection-free equilibrium point:

(9) $$\begin{array}{c} R_{0}=\frac{\lambda }{\left(\mu +\delta \right)N\langle k\rangle }\overset{n}{\underset{i=1}{\sum }}k_{i}^{2}N_{i}^{2}. \end{array}$$

**Proof**

Let *i* = 1 and put it into (3); the following equation can be obtained:

(10) $$\begin{array}{c} \frac{dS_{1}\left(t\right)}{dt}=\mu N_{1}-\mu S_{1}\left(t\right)-\lambda k_{1}S_{1}\left(t\right)\Theta \left(t\right)+\delta I_{1}\left(t\right),\\ \frac{dI_{1}\left(t\right)}{dt}=\lambda k_{1}S_{1}\left(t\right)\Theta \left(t\right)-\mu I_{1}\left(t\right)-\delta I_{1}\left(t\right). \end{array}$$

Two equations from (10) are given the partial derivative with the aspects of *S*_1_, *I*_1_, *S*_2_, *I*_2_,…, *S*_*n*_, *I*_*n*_ and then set *I*_*i*_ = 0. *A*2 × 2*n* dimensional matrix is obtained, where *g*(*j*) = *k*_*j*_ *P*(*k*_*j*_)/〈*k*〉, *j* = 1,2,…, *n*:

(11) $$\begin{array}{c} \left(\begin{array}{cccccccc} -\mu & -\lambda k_{1}S_{1}g\left(1\right) & \cdots & 0 & -\lambda k_{1}S_{1}g\left(j\right) & \cdots & 0 & -\lambda k_{1}S_{1}g\left(n\right)\\ 0 & -\left(\mu +\delta \right)+\lambda k_{1}S_{1}g\left(1\right) & \cdots & 0 & \lambda k_{1}S_{1}g\left(j\right) & \cdots & 0 & \lambda k_{1}S_{1}g\left(n\right) \end{array}\right). \end{array}$$

Similarly, when *i* = 2,3,…, *n*, we take the derivative of formula (3) with the aspects of *S*_1_, *I*_1_,…, *S*_*n*_, *I*_*n*_ and then set *I*_*i*_ = 0. With matrix (11), a 2*n* × 2*n* dimensional matrix is obtained:

(12) $$\begin{array}{c} \left(\begin{array}{cccccccc} -\mu & -\lambda k_{1}S_{1}g\left(1\right) & \cdots & 0 & -\lambda k_{1}S_{1}g\left(j\right) & \cdots & 0 & -\lambda k_{1}S_{1}g\left(n\right)\\ 0 & -\left(\mu +\delta \right)+\lambda k_{1}S_{1}g\left(1\right) & \cdots & 0 & \lambda k_{1}S_{1}g\left(j\right) & \cdots & 0 & \lambda k_{1}S_{1}g\left(n\right)\\ \vdots & \vdots & & \vdots & \vdots & & \vdots & \vdots \\ 0 & -\lambda k_{j}S_{j}g\left(1\right) & \cdots & -\mu & -\lambda k_{j}S_{j}g\left(j\right) & \cdots & 0 & -\lambda k_{j}S_{j}g\left(n\right)\\ 0 & \lambda k_{j}S_{j}g\left(1\right) & \cdots & 0 & -\left(\mu +\delta \right)+\lambda k_{j}S_{j}g\left(j\right) & \cdots & 0 & \lambda k_{j}S_{j}g\left(n\right)\\ \vdots & \vdots & & \vdots & \vdots & & \vdots & \vdots \\ 0 & -\lambda k_{n}S_{n}g\left(1\right) & \cdots & 0 & -\lambda k_{n}S_{n}g\left(j\right) & & -\mu & -\lambda k_{n}S_{n}g\left(n\right)\\ 0 & \lambda k_{n}S_{n}g\left(1\right) & \cdots & 0 & \lambda k_{n}S_{n}g\left(j\right) & & 0 & -\left(\mu +\delta \right)+\lambda k_{n}S_{n}g\left(n\right) \end{array}\right). \end{array}$$

According to [33], *n* eigen values of matrix (12) are all equal to −*u*. Lines or columns including any of these *n* eigen values are removed, and a *n* × *n* dimensional matrix is obtained:

(13) $$\begin{array}{c} \left(\begin{array}{cccccc} -\left(\mu +\delta \right)+\lambda k_{1}S_{1}g\left(1\right) & \lambda k_{1}S_{1}g\left(2\right) & \cdots & \lambda k_{1}S_{1}g\left(j\right) & \cdots & \lambda k_{1}S_{1}g\left(n\right)\\ \lambda k_{2}S_{2}g\left(1\right) & -\left(\mu +\delta \right)+\lambda k_{2}S_{2}g\left(2\right) & & \lambda k_{2}S_{2}g\left(j\right) & & \lambda k_{2}S_{2}g\left(n\right)\\ \vdots & \vdots & & \vdots & & \vdots \\ \lambda k_{j}S_{j}g\left(1\right) & \lambda k_{j}S_{j}g\left(2\right) & & -\left(\mu +\delta \right)+\lambda k_{j}S_{j}g\left(j\right) & & \lambda k_{j}S_{j}g\left(n\right)\\ \vdots & \vdots & & \vdots & & \vdots \\ \lambda k_{n}S_{n}g\left(1\right) & \lambda k_{n}S_{n}g\left(2\right) & & \lambda k_{n}S_{n}g\left(j\right) & & -\left(\mu +\delta \right)+\lambda k_{n}S_{n}g\left(n\right) \end{array}\right). \end{array}$$

A series of transformations for matrix (13) are performed, and then the following matrix is given:

(14) $$\begin{array}{c} \left(\begin{array}{cccccc} -\left(\mu +\delta \right) & 0 & \cdots & 0 & \cdots & \lambda k_{1}S_{1}g\left(n\right)\\ 0 & -\left(\mu +\delta \right) & \cdots & 0 & \cdots & \lambda \left(k_{1}S_{1}g\left(1\right)+k_{2}S_{2}g\left(2\right)\right)\frac{g\left(n\right)}{g\left(2\right)}\\ \vdots & \vdots & & \vdots & & \vdots \\ 0 & 0 & \cdots & -\left(\mu +\delta \right) & \cdots & \lambda \overset{j}{\underset{i=1}{\sum }}\left[k_{i}S_{i}g\left(i\right)\right]\frac{g\left(n\right)}{g\left(j\right)}\\ \vdots & \vdots & & \vdots & & \vdots \\ 0 & 0 & \cdots & 0 & \cdots & -\left(\mu +\delta \right)+\lambda \overset{n}{\underset{i=1}{\sum }}\left[k_{i}S_{i}g\left(i\right)\right] \end{array}\right). \end{array}$$

Obviously, the matrix (14) has an upper triangular one, and its characteristic equation is as follows:

(15) $$\begin{array}{c} {\left[\lambda +(\mu +\delta )\right]}^{n-1}\cdot \left[\lambda +\left(\mu +\delta \right)-\lambda \overset{n}{\underset{i=1}{\sum }}\left[k_{i}S_{i}g\left(i\right)\right]\right]=0. \end{array}$$

From (15), the characteristic values are obtained:

(16) $$\begin{array}{c} \lambda_{1}=-\left(\mu +\delta \right),\;\;\lambda_{2}=-\left(\mu +\delta \right)+\lambda \overset{n}{\underset{i=1}{\sum }}\left[k_{i}S_{i}g\left(i\right)\right]. \end{array}$$

According to Routh-Hurwitz criterion, if and only if all of characteristic values are less than zero, the propagation system will eventually be stable at the equilibrium point *E*_0_. Obviously, *λ*_1_ are negative and the stability relies on *λ*_2_. If *λ*_2_ is less than 0, the equilibrium will be achieved. By transformation, the stability condition is derived as

(17) $$\begin{array}{c} R_{0}=\frac{\lambda }{\left(\mu +\delta \right)N\langle k\rangle }\overset{n}{\underset{i=1}{\sum }}k_{i}^{2}N_{i}^{2}<1. \end{array}$$

The proof is complete.

**Corollary 2 .**

When the degree of a mobile node grows, the basic reproduction number *R*_0_ gets increased, which means that it increases difficulty in realizing the stability for the propagation system of the mobile phone virus.

**Proof**

Equation (17) can be converted into the following inequality:

(18) $$\begin{array}{c} R_{0}=\frac{\lambda }{\left(\mu +\delta \right)N\langle k\rangle }\cdot \frac{\sum_{i=1}^{n}k_{i}^{2}N_{i}^{2}}{\sum_{i=1}^{n}k_{i}N_{i}}<1. \end{array}$$

Obviously, ∑_*i*=1_^*n*^ *k*_*i*_^2^ *N*_*i*_^2^/∑_*i*=1_^*n*^ *k*_*i*_ *N*_*i*_ is a monotonic function of *k*_*i*_. When the degree *k*_*i*_ of the mobile phone node is increased, *R*_0_ will also grow. It makes (18) more difficult to be satisfied.  Corollary 2 is thus drawn.

## 4. Modeling the Propagation of the Vulnerability-Exploiting Mobile Phone Virus

### 4.1. M-SIR Model

According to the development of virus, the mobile virus will eventually become a mobile worm which is called vulnerability-exploiting virus. The vulnerability-exploiting virus will automatically propagate by exploiting vulnerabilities existing in mobile phone operating systems or application software. Patching can be applied to repair vulnerabilities and then protect mobile phones from attacks. According to the spread property of vulnerability-exploiting virus, the epidemic state of a node is divided as follows: susceptible state (*S*), infectious state (*I*), and recovered state (*R*).

Susceptible state (*S*): nodes have not been infected by any user-tricking virus but are prone to infection. Infectious state (*I*): nodes have been infected by vulnerability-exploiting virus and they may infect some nodes in state S. Recovered state (*R*): nodes are cleaned of vulnerability-exploiting virus and immune to the same type of cleaned virus.

**Figure 2:**
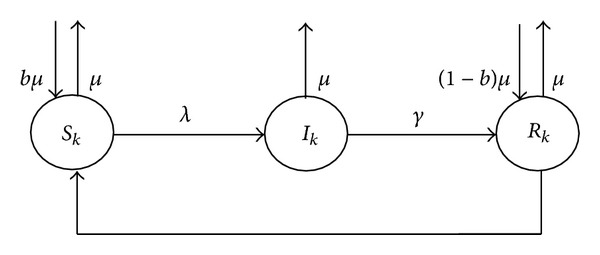
State transition graph of the *k*th kind of nodes in M-SIR model.

The propagation modeling of vulnerability-exploiting mobile phone virus under complex network called M-SIR model is proposed, which means mobile phone virus SIR (susceptible-infectious-recovered) propagation model. The state transforming process of any kind of nodes in M-SIR model is illustrated in Figure 2.

In the M-SIR model, a node in the *k*th kind can transit to the infectious state if it is at the susceptible state. The infection probability is presented by *λ*. The infectious node can clean the virus through patching with the immune rate *γ*. Once patched, the mobile is immune to the virus permanently. The susceptible node can also be patched in advance of infection with patching rate *ω* and transits to the recovered state. To maintain the balance of the network system, the “death” rate and the “birth” rate are all *μ*. The “new born” mobiles are all in the susceptible state. But the “new born” mobiles become not only susceptible ones but also “immune” ones, because new mobiles may install new versions of software with patches. The description of related parameters in M-SIR model is shown in Table 2.

Define *R*_*i*_(*t*) as the number of the *i*th kinds of immune mobiles at time *t*. A mobile can be in one of three states for a time, and the sum for three classes of mobiles is as

(19) $$\begin{array}{c} S_{i}\left(t\right)+I_{i}\left(t\right)+R_{i}\left(t\right)=N_{i}. \end{array}$$

According to the above analysis and state transition graph in Figure 2, given a topology of a social network, the number of susceptible, infected, and recovered nodes of the *i*th kind at time *t* in the M-SIR model can be presented by

(20) $$\begin{array}{c} \frac{dS_{i}\left(t\right)}{dt}=b\mu N_{i}-\mu S_{i}\left(t\right)-\lambda k_{i}S_{i}\left(t\right)\Theta \left(t\right)-\omega S_{i}\left(t\right),\\ \frac{dI_{i}\left(t\right)}{dt}=\lambda k_{i}S_{i}\left(t\right)\Theta \left(t\right)-\mu I_{i}\left(t\right)-\gamma I_{i}\left(t\right),\\ \frac{dR_{i}\left(t\right)}{dt}=\left(1-b\right)\mu N_{i}+\gamma I_{i}\left(t\right)-\mu R_{i}\left(t\right). \end{array}$$

There are *n* kinds of nodes in the network, so the differential equations of the M-SIR model can be concluded as the following equation:

(21) $$\begin{array}{c} \frac{dS\left(t\right)}{dt}=\overset{n}{\underset{i=1}{\sum }}\frac{dS_{i}\left(t\right)}{dt},\\ \frac{dI\left(t\right)}{dt}=\overset{n}{\underset{i=1}{\sum }}\frac{dI_{i}\left(t\right)}{dt},\\ \frac{dR\left(t\right)}{dt}=\overset{n}{\underset{i=1}{\sum }}\frac{dR_{i}\left(t\right)}{dt}. \end{array}$$

### 4.2. Infection-Free Equilibrium Point

In order to obtain the infection-free equilibrium point, (20) is converted into the following equation:

(22) $$\begin{array}{c} b\mu N_{i}-\mu S_{i}\left(t\right)-\lambda k_{i}S_{i}\left(t\right)\Theta \left(t\right)-\omega S_{i}\left(t\right)=0,\\ \lambda k_{i}S_{i}\left(t\right)\Theta \left(t\right)-\mu I_{i}\left(t\right)-\gamma I_{i}\left(t\right)=0,\\ \left(1-b\right)\mu N_{i}+\gamma I_{i}\left(t\right)-\mu R_{i}\left(t\right)=0. \end{array}$$

Solving (22), *S*_*i*_(*t*), *I*_*i*_(*t*), and *R*_*i*_(*t*) are derived as follows:

(23) $$\begin{array}{c} S_{i}=\frac{b\mu }{\mu +\omega }N_{i},\\ I_{i}=0,\\ R_{i}=N_{i}-\frac{b\mu }{\mu +\omega }. \end{array}$$

There are total *n* kinds of nodes. Each kind of nodes has an infection-free equilibrium point. So the infection-free equilibrium point of the mobile phone virus propagation system under the M-SIR model is *E*_1_*(*S*_1_, 0, *R*_1_, *S*_2_, 0, *R*_2_ …, *S*_*n*_, 0, *R*_*n*_), where

(24) $$\begin{array}{c} S_{i}=\frac{b\mu }{\mu +\omega }N_{i},\;\;I_{i}=0,\;\;R_{i}=N_{i}-\frac{b\mu }{\mu +\omega }. \end{array}$$

### 4.3. Stability of the Infection-Free Equilibrium

**Theorem 3 .**

If the basic reproduction number *R*_1_ < 1, the mobile phone virus propagation system under the M-SIR model will stabilize at the infection-free equilibrium point:

(25) $$\begin{array}{c} R_{1}=\frac{\lambda b\mu }{\left(\mu +\gamma \right)\left(\mu +\omega \right)N\langle k\rangle }\cdot \overset{n}{\underset{i=1}{\sum }}i^{2}N_{i}^{2}. \end{array}$$

**Proof**

Take the partial derivative of three equations to the right in (20) with the aspects of *S*_1_, *I*_1_, *S*_2_, *I*_2_,…, *S*_*n*_, *I*_*n*_. With *I*_*i*_ = 0, a 2*n* × 2*n* dimensional matrix is given:

(26) $$\begin{array}{c} \left(\begin{array}{cccccccc} -\mu -\omega & -\lambda k_{1}S_{1}g\left(1\right) & \cdots & 0 & -\lambda k_{1}S_{1}g\left(j\right) & \cdots & 0 & -\lambda k_{1}S_{1}g\left(n\right)\\ 0 & -\left(\mu +\gamma \right)+\lambda k_{1}S_{1}g\left(1\right) & \cdots & 0 & \lambda k_{1}S_{1}g\left(j\right) & \cdots & 0 & \lambda k_{1}S_{1}g\left(n\right)\\ \vdots & \vdots & & \vdots & \vdots & & \vdots & \vdots \\ 0 & -\lambda k_{j}S_{j}g\left(1\right) & \cdots & -\mu -\omega & -\lambda k_{j}S_{j}g\left(j\right) & \cdots & 0 & -\lambda k_{j}S_{j}g\left(n\right)\\ 0 & \lambda k_{j}S_{j}g\left(1\right) & \cdots & 0 & -\left(\mu +\gamma \right)+\lambda k_{j}S_{j}g\left(j\right) & \cdots & 0 & \lambda k_{j}S_{j}g\left(n\right)\\ \vdots & \vdots & & \vdots & \vdots & & \vdots & \vdots \\ 0 & -\lambda k_{n}S_{n}g\left(1\right) & \cdots & 0 & -\lambda k_{n}S_{n}g\left(j\right) & & -\mu -\omega & -\lambda k_{n}S_{n}g\left(n\right)\\ 0 & \lambda k_{n}S_{n}g\left(1\right) & \cdots & 0 & \lambda k_{n}S_{n}g\left(j\right) & & 0 & -\left(\mu +\gamma \right)+\lambda k_{n}S_{n}g\left(n\right) \end{array}\right), \end{array}$$

where *g*(*j*) = *k*_*j*_ *P*(*k*_*j*_)/〈*k*〉, *j* = 1,2,…, *n*.

Removing the lines and columns including −*μ* + *ω*, a matrix of *n* × *n* dimensional is given as follows:

(27) $$\begin{array}{c} \left(\begin{array}{cccccc} -\left(\mu +\gamma \right)+\lambda k_{1}S_{1}g\left(1\right) & \lambda k_{1}S_{1}g\left(2\right) & \cdots & \lambda k_{1}S_{1}g\left(j\right) & \cdots & \lambda k_{1}S_{1}g\left(n\right)\\ \lambda k_{2}S_{2}g\left(1\right) & -\left(\mu +\gamma \right)+\lambda k_{2}S_{2}g\left(2\right) & & \lambda k_{2}S_{2}g\left(j\right) & & \lambda k_{2}S_{2}g\left(n\right)\\ \vdots & \vdots & & \vdots & & \vdots \\ \lambda k_{j}S_{j}g\left(1\right) & \lambda k_{j}S_{j}g\left(2\right) & & -\left(\mu +\gamma \right)+\lambda k_{j}S_{j}g\left(j\right) & & \lambda k_{j}S_{j}g\left(n\right)\\ \vdots & \vdots & & \vdots & & \vdots \\ \lambda k_{n}S_{n}g\left(1\right) & \lambda k_{n}S_{n}g\left(2\right) & & \lambda k_{n}S_{n}g\left(j\right) & & -\left(\mu +\gamma \right)+\lambda k_{n}S_{n}g\left(n\right) \end{array}\right). \end{array}$$

The second column of the matrix (26) multiplying by −*g*(1)/*g*(2) is added to the first column, and then the third column multiplying by −*g*(2)/*g*(3) is added to the second column and so on. After that, the first row multiplying by *g*(1)/*g*(2) is added to the second row, and then the second row multiplying by *g*(2)/*g*(3) is added to the third row and so on. The following matrix is thus obtained:

(28) $$\begin{array}{c} \left(\begin{array}{cccccc} -\left(\mu +\gamma \right) & 0 & \cdots & 0 & \cdots & \lambda k_{1}S_{1}g\left(n\right)\\ 0 & -\left(\mu +\gamma \right) & & 0 & & \lambda \left[k_{1}S_{1}g\left(1\right)+2k_{2}S_{2}g\left(2\right)\right]\frac{g\left(n\right)}{g\left(2\right)}\\ \vdots & \vdots & & \vdots & & \vdots \\ 0 & 0 & & -\left(\mu +\gamma \right) & & \lambda \overset{j}{\underset{i=1}{\sum }}\left[k_{i}S_{i}g\left(i\right)\right]\frac{g\left(n\right)}{g\left(j\right)}\\ \vdots & \vdots & & \vdots & & \vdots \\ 0 & 0 & & 0 & & -\left(\mu +\gamma \right)+\lambda \overset{n}{\underset{i=1}{\sum }}\left[k_{i}S_{i}g\left(i\right)\right] \end{array}\right). \end{array}$$

The characteristic equation of (28) is showed as follows:

(29) $$\begin{array}{c} {\left[\lambda +(\mu +\delta )\right]}^{n-1}\cdot \left[\lambda +\left(\mu +\delta \right)-\lambda \overset{n}{\underset{i=1}{\sum }}\left[k_{i}S_{i}g\left(i\right)\right]\right]=0. \end{array}$$

The characteristic values are as follows:

(30) $$\begin{array}{c} \lambda_{1}=-\left(\mu +\delta \right),\;\;\lambda_{2}=-\left(\mu +\delta \right)+\lambda \overset{n}{\underset{i=1}{\sum }}\left[k_{i}S_{i}g\left(i\right)\right]. \end{array}$$

According to Routh-Hurwitz criterion, if and only if all of the characteristic values are less than zero, the propagation system will eventually be stable at the equilibrium point *E*_1_*. By transformation of *λ*_2_, the stability condition is obtained as

(31) $$\begin{array}{c} R_{1}=\frac{\lambda \sum_{i=1}^{n}\left[iS_{i}g\left(i\right)\right]}{\mu +\gamma }=\frac{\lambda b\mu }{\left(\mu +\gamma \right)\left(\mu +\omega \right)N\langle k\rangle }\cdot \overset{n}{\underset{i=1}{\sum }}i^{2}N_{i}^{2}<1. \end{array}$$

The proof is complete.

## 5. Constructing the Network Topology

The attacks target of the mobile virus is to infect the smart phone. The propagation path of mobile virus obeys the mobile user's social network, which has its own characters and greatly affects the propagation of the mobile phone virus. Thus it is indispensable to construct such a network to simulate the propagation of the mobile phone virus and validate our models.

The social network which is the propagation environment of mobile virus is a typical complex network. In the real world lots of networks have been proved to be complex network such as World Wide Web and email. The complex network has the following two characteristics: the degree of a node follows the power-law distribution and the network appears as small-world phenomenon. It is hard to put the real mobile virus into the real mobile network. So network topology generator called Inet3.0 is used to create a complex network to simulate the environment of mobile virus.

Inet is a topology generator developed by the University of Michigan and its current version has been upgraded to 3.0. When giving the total number of *N* nodes, Inet3.0 could output the information of *N* nodes including the position, degree, and the neighbors. Inet3.0 simulates the topology structure of the Internet and it accords with the characteristics of the complex network. Firstly, nodes' degrees generated by Inet3.0 follow the power-law distribution. Secondly, the characteristic path length created by Inet3.0 is short, which reflects the effect of the small-world phenomenon of social network. However, the clustering coefficient of the network built by Inet3.0 is relatively large. The network generated by Inet3.0 is much closed to the complex network and can be applied for simulating the propagation of the mobile phone virus.

**Figure 3:**
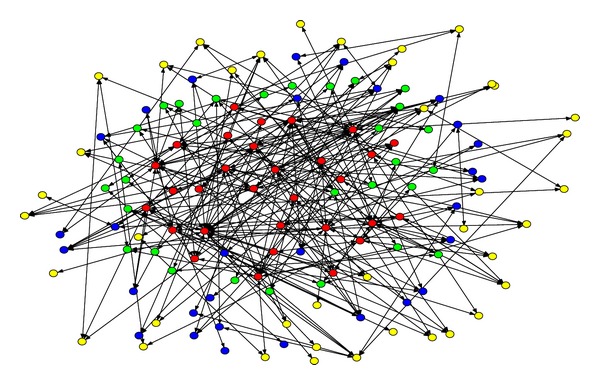
The distribution and connectivity of 130 nodes in the network generated by Inet3.0.

In this paper, Inet3.0 is used to build a complex network which contains 10000 nodes. There are 118 different kinds of degrees among which the biggest value is 1799 and the least one is 1. Due to the high density of the topology and the page limit, it is difficult to differentiate the connectivity between nodes.  Figure 3 shows the distribution and the connectivity of only 130 nodes in the topology structure, and the degrees of them are the biggest of all 10000 nodes.

Among the 130 nodes, the 30 red nodes are those with the biggest degrees; the 30 green ones are those with bigger degrees; the 30 blue ones are those with smaller degrees; the 40 yellow ones are those with the smallest degrees.

## 6. Numerical and Simulation Experiments

To verify the accuracy of theoretical analysis and the correctness of both M-SIS and M-SIR models, the numerical and simulation experiments are separately carried out. Numerical experiments are based on iterations of formulae and can directly reflect the property of the models. It is hard to simulate the real propagation environment of mobile phones virus. So the simulation experiments are carried out like other researchers [22–30]. Our simulation is a discrete-time simulation and well embodies the propagation of viruses in which node data are obtained on a time interval every second. Different from numerical experiments, the simulation imitates the real environment and is more closed to reality.

To raise the accuracy, the experiments under the same condition are carried out for 100 times, and the experiment result is derived from the average of 100 results.  Algorithm 1 is the algorithm of the simulation which embodies the topology of the network. It is noted that one susceptible mobile can only be infected by its neighboring infected mobiles. The two-dimensional array Link_Matrix [][] is used to store the joined relationship between nodes.

### 6.1. Experiment for the M-SIS Model

The parameters in our experiments are chosen based on the research results of Zou et al. [34] and Wang et al. [35]. Due to the limit of computer memory and Inet3.0, 10,000 mobile phone nodes are set in our network system.

The contact infection rate *λ* of the mobile phone virus is set at 0.00003 with the same magnitude of the initial infection rate in Zou et al.'s research [34]. Similarly, the death/birth rate *μ* is assigned to be 0.00002 based on Wang et al.'s study [35]. The recovery rate *δ* is assumed to set 0.1. At the beginning, the mobile phone virus spreads along the edges of mobile phone nodes which own few contacts with others and then attacks core nodes. Therefore, there are 10 infected mobile phones with the degree of 1 initially, which means that the initial infected nodes only have one contact with other nodes.

**Figure 4:**
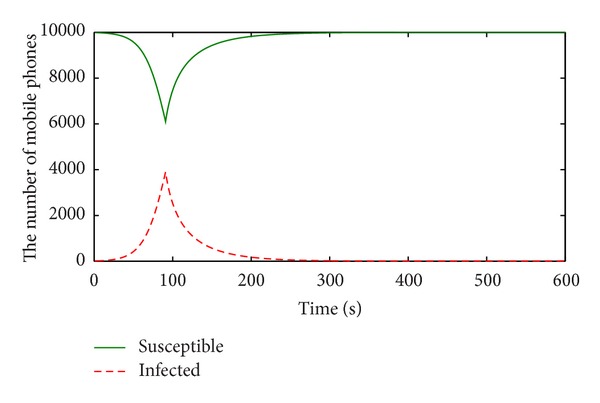
Numerical results in the M-SIS model.

The numerical results of the number of susceptible, infected mobile phones over time in M-SIS model are showed, respectively, in Figure 4.

To observe the propagation of the mobile user-tricking virus, virus-killing measure is taken after the 90 s, and sharp points appear in the curves at 90 s.

According to Theorem 1, the basic reproduction number *R*_0_ is about 0.8 with the above parameters. It means that the propagation system of the mobile user-tricking virus under the M-SIS model will be eventually stable at its infection-free equilibrium point. Obviously, the number of infected mobile phones shrinks to 0 and that of susceptible ones is up to 10000 in Figure 4, which indicates that the infection-free equilibrium is achieved. The accuracy of theoretical analysis gets verified.

**Figure 5:**
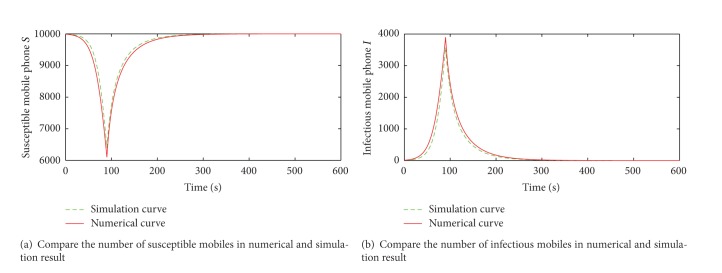
Comparison of numerical and simulation result.

To check the correctness of M-SIS propagation model, the simulation experiments have been executed and the simulation results are compared with numerical results under the same parameters as shown in Figure 5.

Figure 5(a) compares the number of susceptible mobiles in numerical and simulation result and Figure 5(b) compares the number of susceptible mobiles. It is seen that the numerical curves match the simulation ones very well, which verifies the correctness of the M-SIS propagation model.

**Figure 6:**
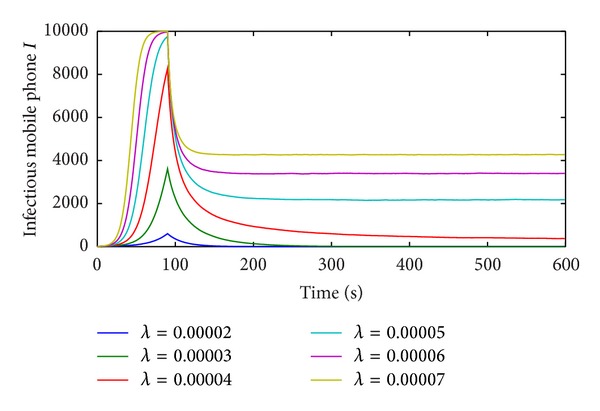
Impact of the contact infection rate *λ* on the M-SIS model.

The affections of different parameters on the mobile user-tricking virus propagation model are tested. The contact infection rate *λ* is firstly discussed. *λ* is specified as 0.00002, 0.00003, 0.00004, 0.00005, 0.00006, and 0.00007, respectively, and other parameters remain unchanged. With different contact infection rates, the propagation trends of the mobile user-tricking virus are showed in Figure 6.

The increase of the contact infection rate can fasten the propagation of mobile user-tricking virus. But when it increases to certain extent, the impact of the mobile user-tricking virus goes down. The larger contact infection rate is, the more nodes are infected. So the contact infection rates can rapid the propagation speed and wide the propagation scope of mobile user-tricking virus.

**Figure 7:**
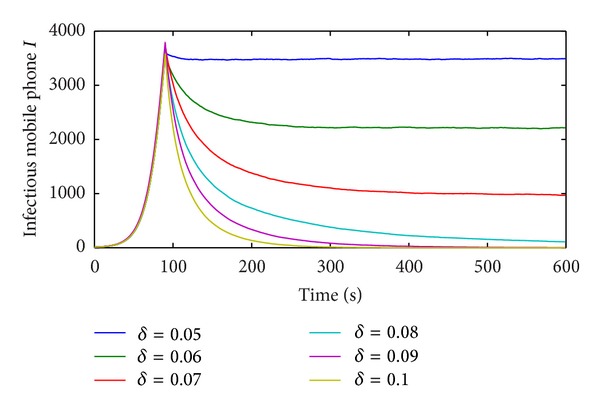
Impact of infection recovery rate *δ* on the M-SIS model.

The infection recovery rate *δ* is also discussed while other parameters remain unchanged. *δ* is set at 0.05, 0.06, 0.07, 0.08, 0.09, and 0.1 respectively, and the propagations trends of the mobile user-tricking virus are given in Figure 7.

With the increase of *δ*, the number of infected nodes decreases, but all the curves reach the peak at the same time. It means that the infection recovery rate can only affect the spread scope of mobile user-tricking virus. It cannot rapid the propagation speed.

### 6.2. Experiment for the M-SIR Model

In this experiment, the patching rate *γ* for infected mobiles is 0.01 based on the research of Wang et al. [35]. The root of mobile vulnerability-exploiting virus existing is software vulnerabilities which are inevitable during the design and implementation process of software and hard to detect. Due to lots of bandwidth consumption the patch cannot be distributed in time, so the patching rate *ω* for susceptible mobile phones is relatively small and is set as 0.0001. And it is assumed that the probability *b* that the “new born” mobile phone becomes susceptible one is 0.6. Other parameters are set the same as the ones in the M-SIS model.

**Figure 8:**
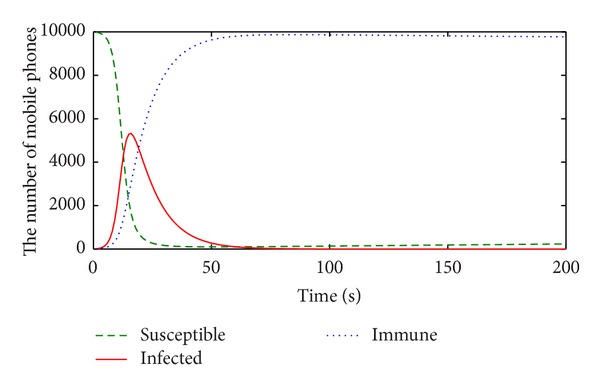
Numerical results in the M-SIR model.

The numerical results of the susceptible, infected, and immune mobile phones in the M-SIR model are given in Figure 8.

All infected mobile phones vanish and the population in the long term is in an immune state. According to Theorem 3, the basic reproduction number *R*_1_ is about 0.8 < 1, which means that the propagation system of mobile phone virus under the M-SIR model will stabilize at its infection-free equilibrium point. In Figure 8 the susceptible, infected, and immune state mobile phones all reach their equilibrium points. This is fully consistent with the conclusions of Theorem 3.

**Figure 9:**
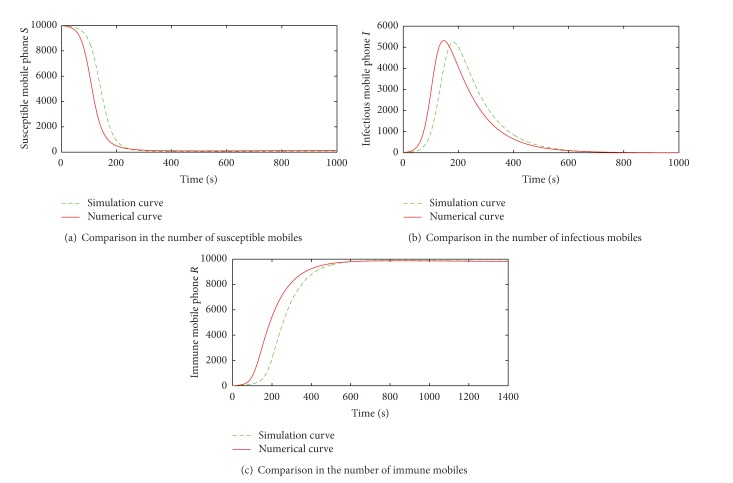
Comparisons between numerical and simulation results in the M-SIR model.

The numerical results and simulation ones in susceptible, infected, and immune mobile phones, respectively, under the M-SIR model are shown in Figure 9.

**Figure 10:**
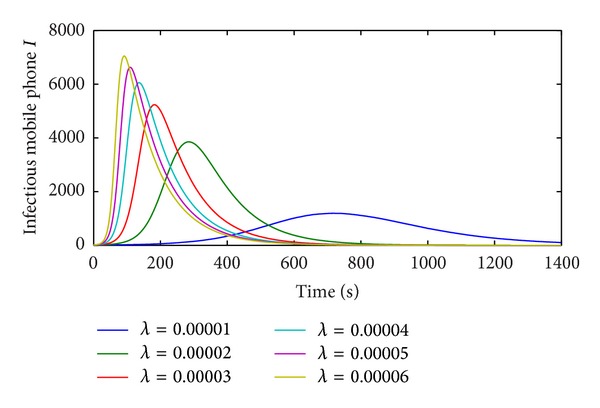
Effect of the contact infection rate *λ* in the M-SIR model.

The simulation curves of all states are almost consistent with the numerical ones which prove the correctness of the M-SIR model. The effect of contact infection rate to the propagation of vulnerability-exploiting mobile phone virus is shown in Figure 10.

Figure 10 shows the propagation trends of vulnerability-exploiting mobile phone virus with six different contact infection rates. With the increasing of the contact infection rate, the spread speed of the vulnerability-exploiting mobile phone virus is promoted, which makes the vulnerability-exploiting mobile phone virus reach the peak with little time. The scope of vulnerability-exploiting mobile phone virus also widens with the higher contact infection rate. The higher contact infection rate is the more nodes are infected. But the impact on the propagation is weakening with *λ* going up to some extent.

**Figure 11:**
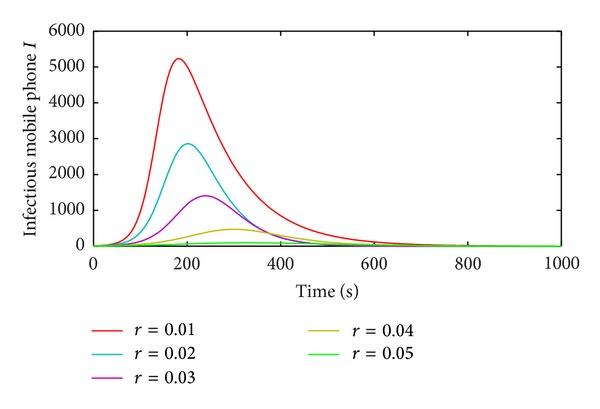
Effect of the immune rate *γ* in the M-SIR model.

The performance of the immune rate to the propagation of vulnerability-exploiting mobile phone virus is discussed in Figure 11.

Figure 11 gives the propagations of the vulnerability-exploiting mobile phone virus with five different immune rates. The immune rate can affect the speed and scope of propagation. Obviously, the more the immune rate *γ* is, the weaker the spread capability of the vulnerability-exploiting mobile phone virus is. Therefore, in order to guarantee normal applications of mobile phones and suppress the propagation speed and the propagation scope of mobile phone virus, we should choose a reasonable value for immune rate *γ*.

## 7. Conclusions

The objective of this paper is to model two kinds of mobile phone virus under two important factors (viz., the characteristics of mobile phone viruses and the network topology structure) and then to find out certain means to suppress the propagation of mobile phone virus. The M-SIS and M-SIR propagation models for mobile phone viruses are proposed, combining with the structural characteristics of the complex network.

The M-SIS propagation model is effective to predict the propagation of the user-tricking mobile phone virus. It reflects the characteristic of the mobile virus, which is difficult to completely remove, and the removed mobile phone virus can reinfect the same mobile phone.

The M-SIR propagation model is suitable to describe the vulnerability-exploiting mobile phone virus. It reflects the characteristic of the mobile virus, which spreads by exploiting vulnerabilities, and the mobile phone can be immune to the mobile phone virus after virus removal and patching.

Through analysis, the stable infection-free equilibrium point and the stability condition of the two propagation models are derived. The basic reproduction numbers *R*_0_ and *R*_1_ are given, which can determine whether the mobile phone virus extinguishes. When *R*_0_ < 1 and *R*_1_ < 1, the proposed M-SIS and M-SIR models have only a worm-free equilibrium, respectively, which is globally stable and implies that the worm dies out eventually. Then some numerical and simulation experiments are carried out which prove that our models are correct and fully consistent with the conclusions of our analysis. Our future work will expand this model which can characterize more features of mobile phone virus, for example, taking delay or impulse into consideration.

## Figures and Tables

**Algorithm 1:**
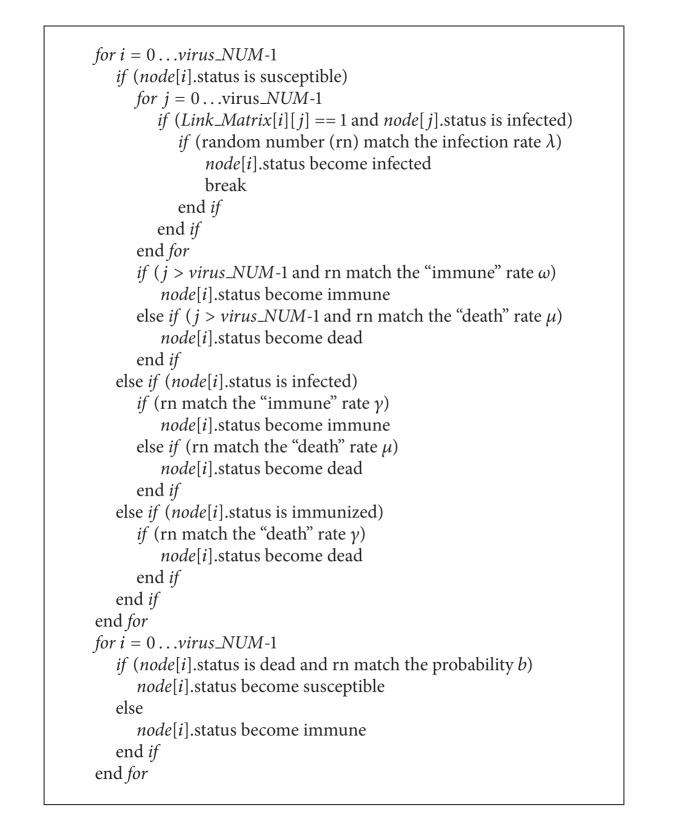


**Table 1:** Parameters description in the M-SIS model.

Parameter	Meaning
*N*	The total number of mobiles
*S*_*i*_(*t*)	The number of the *i*th kinds of mobiles in the susceptible state at time *t*
*I*_*i*_(*t*)	The number of the *i*th kinds of mobiles in the infectious state at time *t*
*N*_*i*_	The number of the *i*th kinds of mobiles at time *t*
*λ*	The contact infection rate
*δ*	The infection recovery rate
*μ*	The “birth/death” rate

**Table 2:** Notations in the M-SIR model.

Parameter	Meaning
*N*	The total number of mobiles
*S*_*i*_(*t*)	The number of the *i*th kinds of mobiles in the susceptible state at time *t*
*I*_*i*_(*t*)	The number of the *i*th kinds of mobiles in the infected state at time *t*
*R*_*i*_(*t*)	The number of the *i*th kinds of mobiles in the “immune” state at time *t*
*N*_*i*_	The number of the *i*th kinds of mobiles at time *t*
*λ*	The contact infection rate
*γ*	The “immune” rate for infected mobiles
*ω*	The “immune” rate for susceptible mobiles
*μ*	The “birth/death” rate
*b*	The probability of new “born” susceptible mobiles
1 − *b*	The probability of new “born” immune mobiles
